# Perception of risk among children: Exploring the risk of TB-rickettsial disease based on the children’s drawing pictures in a Mayan community of Yucatan

**DOI:** 10.34172/hpp.2023.16

**Published:** 2023-07-10

**Authors:** Karla Dzul-Rosado, Teresita Castillo-León, Elisia Montalvo-Nah, Juan Arias-León, Fernando Puerto-Manzano

**Affiliations:** ^1^Autonomous University of Yucatan, Regional Research Center “Dr. Hideyo Noguchi”, Laboratory of Emerging and Reemerging Diseases, Mérida, Yucatán, México; ^2^Autonomous University of Yucatan, Faculty of Psychology, Mérida, Yucatán, México; ^3^Autonomous University of Yucatan, Faculty of Medicine Mérida Yucatán, México

**Keywords:** TBD, Rickettsiosis, Drawings, Social cognitive, Beliefs model

## Abstract

**Background:**

It is important to work on designs of health promotion strategies that involve educational interventions about the risk factors associated to TB-rickettsiosis (Tick-Borne rickettsiosis). Children’s drawings provide a window into their thoughts and feelings. The aim was to analyze children’s risk perceptions regarding by the presence of ticks in a rural community in southeast Mexico.

**Methods:**

The main framework used was a social cognitive perspective under the Health Belief Model. Study was carried out in rural elementary schools and included a drawing contest. A total of 224 children (8-12 years old) participated. Drawings were coded and classified; descriptive trend analysis was performed using counts and percentages. The qualitative data was analyzed by researcher experts in TB- rickettsiosis and using Atlas ti V.8.

**Results:**

Results are presented in seven categories that consider environmental elements, actors, and cognitive aspects. The environmental context, 99.1% of the drawings represent sites outside the home (92% include vegetation with the presence of ticks in their immediate external environment). In the actors’ category, 69.6% included people carrying out activities (prevention and risk). As for the cognitive aspects, 70% included domestic pets and 13.4% farmyards animals.

**Conclusion:**

The children expressed their ideas about risk factors and preventive measures against TB-rickettsiosis with a self-care approach. They acquired knowledge about preventive strategies and clinical symptoms. It is necessary to evaluate what happens after an intervention and how they implement in their lives what they have learned.

## Introduction

 A number of tick-borne diseases in humans have increased their incidence and geographic range over the past few decades, and there is concern that they will pose an even greater threat to public health in the future.^1^ Various health agencies and institutions are currently working on the design of health promotion strategies that involve educational interventions, primarily aimed at informing communities about the importance of addressing the risk factors associated with tick borne disease (TBD) s in order to promote self-care.^2-4^ One TBD of epidemiological importance for public health in tropical and subtropical regions, given its re-emergence, is Rickettsiosis, which involves vectors such as fleas, lice and ticks.^5^ One of the most vulnerable groups and, nevertheless, one of the most important in the field of health education, are children.^6^ However, little research has been conducted among this population group and the development of strategies tailored to this age group is limited, so little is known about their perception and management of the problem and the impact that an educational intervention on TB-rickettsiosis could have on them.^7^ Hence the importance of conducting research with children. In this regard, Driesnack concludes that children know more than adults assume, and when their ideas and perceptions are taken into account and they are provided with relevant information, they show a great ability to become even more involved. However, research and intervention actions have recently been directed more at primary school-age children.^8^ Moreover, there has been documentation of the use of the Health Belief Model (HBM) among school children to create an environment which furthers prevention of tick-borne diseases such as Lyme disease.^8^ In this context, educational and psychological research has used different methodological tools to study children’s conceptions of various phenomena.^9^ Among these tools are questionnaires, surveys, structured and semi-structured interviews and schematic representations, but these are often designed based on their applications with adults, and therefore tend to be biased when applied to children.^10^ This is why in recent years there has been increased interest in the use of children’s drawings, as they facilitate communication skills, particularly regarding events or concepts that would otherwise be difficult to describe.^11-14^ As a technique for exploring ideas, drawing taps holistic understanding and prevents children from feeling constrained by trying to match their knowledge with that of the researcher.^13^ It is also a useful alternative form of expression for children who have difficulty expressing their thoughts verbally.^14^

 Several investigations have used drawing as a research tool with children to identify their perceptions of issues such as: (a) child stigmatization of those who have been affected by the HIV virus^15^; (b) children’s perception of tick-associated diseases^8,16,17^; (c) triatomines and Chagas disease^18,19^; and (d) more recently, issues such as SARS-CoV-2.^20^ Considering the above, the present work has two purposes: (a) to report the main results regarding the perceptions that children have about ticks and their possible health risks, based on an educational intervention. And (b) to document the results of an intervention experience using techniques such as drawing with this age group.

###  Theoretical-methodological framework

 With his cognitive-social theory, Bandura moves away from deterministic positions regarding where the causes of behavior are located, avoiding both the environmentalist and intrapersonal extremes, but rather considering that both aspects interrelate to influence an individual’s behavior.^21^ Hence the importance of taking into account the environmental context itself, as well as the actors within it (particularly regulations and the opinions of significant people), together with what one believes should be done and one’s own behavior. From this interplay of elements, people learn both directly and indirectly (by observation). From the above, we can see how behavior can be influenced by environmental aspects, but also by cognitive aspects, the latter having a great weight. In this context, when we talk about behaviors related to vector-borne diseases, the cognitive aspect affects, particularly beliefs about the perception of risk, which become fundamental. To understand the role of these beliefs, it is very useful to revisit the HBM used as part of an approach to health promotion.^22^ The model addresses perceived severity, perceived susceptibility/vulnerability, the benefits of preventive behaviors, and the obstacles that may prevent adequate care of the problem.

###  Drawing

 Currently, there is widespread interest in the use of art in research: for example, using drawings.^18,22^ This technique is very useful since it is a clear indicator of the child’s motor and mental development, and of their level of learning and creativity. It allows the children greater freedom of expression with their own language, to externalize their moods, the way they see themselves and their perceptions of certain situations in their daily life.^14^ In individual drawings, schoolchildren try to make sense of the world around them.^10^

## Materials and Methods

###  Study design

 An educational intervention study was carried out in a rural community in southeast Mexico. It included three activities: participatory workshops, individual interviews and recreational sessions. It also included a drawing contest for children called My House and the Peech (“tick”, in Maya).

###  Study area

 This intervention was carried out in the community of Teabo, Yucatan, in southeast Mexico; located between N 20° 19’ and 20° and W 89° 11’ and 89° 20’. The district population is 6921 people distributed among 1380 households which are occupying an area of 26 187 km^2^. 50.2% are women and 49.8% are men. The age ranges that concentrated the largest population were 0 to 4 years (716 inhabitants), 5 to 9 years (698 inhabitants) and 20 to 24 years (675 inhabitants). Among them, they concentrated 30.2% of the total population. There is only one public health center. This community has similar socioeconomic and housing conditions to the rest of the towns and villages in the state where cases of rickettsiosis have occurred.

###  Participants

 224 Children from fourth to sixth grade (8-12 years old) from elementary schools in the community of Teabo, Yucatan voluntary participated in an educational intervention to prevent TB-rickettsiosis. They were invited to a drawing contest called “My house and the Peech”. It was important to work with as most children as possible who were studying in elementary schools in the community. We chose 4th to 6th grade because they were more able to speak and write in Spanish and, at this stage of life, children have greater mental maturity and acquire a sense of responsibility while developing their independence. ^23^

###  Procedure

 The educational intervention consisted of three main activities: participatory workshops, individual interviews and play activities. These addressed various topics such as (a) the characteristics of ticks (taxonomy, diversity of species and what they feed on), (b) places where they are usually found (on an animal, free-living, inside and outside the home), (c) animals on which they have been seen (looking for urban, rural or anthropozoonotic life cycles), and (d) risk to humans (if they have been bitten, site of the bite, symptoms they presented after a bite). This intervention was rounded off with an evaluation carried out through a mixed research process that included focus groups and pre- and post-test analysis of the intervention using questionnaires. For the application of these activities, the following procedure was followed:

1. A research team was trained.
2. School teachers and principals were informed about the activity.
3. Children were invited to participate in a drawing contest, called “My house and the *Peech*”, in which they were asked to express individually the knowledge acquired regarding the risk of tick-associated diseases.
4. Students were asked to use a 50 × 65 cm poster size sheet of paper of any color for their drawings. Each student was given two weeks to create a drawing on the topic of *My House and the Peech*(as ticks are generally referred to in Maya), which responded to the questions: 1) What are the places where you think ticks might be? and 2) What actions would you take to prevent ticks? They were asked to write their name, gender and age on the drawings, along with the name of their school and their grade. Material for the drawings was provided for them. No further instructions were given, so children were free to associate ticks with any situation or characteristics they chose. They were asked to produce the drawings in their own homes and bring them back to school. After they were collected, all drawings had an identification number assigned.
5. Finally, a ceremony was held in the community for the presentation of prizes and an exhibition of the drawings. This event raised additional awareness about TB-rickettsiosis in the community.

###  Analysis of the data

 The drawings were converted into digital files, and the elements present were classified and coded. For the analysis of the drawings, we conducted a descriptive analysis to identify trends using counts and percents. “Researcher Triangulation” by TBD experts was used. When there were major discrepancies, the drawings were collectively re-examined; frequencies of presence/absence of the various different themes and sub-themes were calculated; and both the most commonly represented features, and some unique features present in the drawings, were identified. The qualitative data was analyzed using ATLAS-ti V.8. and a primary document was generated with the main ideas. References were created and related to the dimensions, creating a code per dimension. Finally, semantic networks were generated, allowing for the addition of “nodes”.

## Results

**Figure 1:**
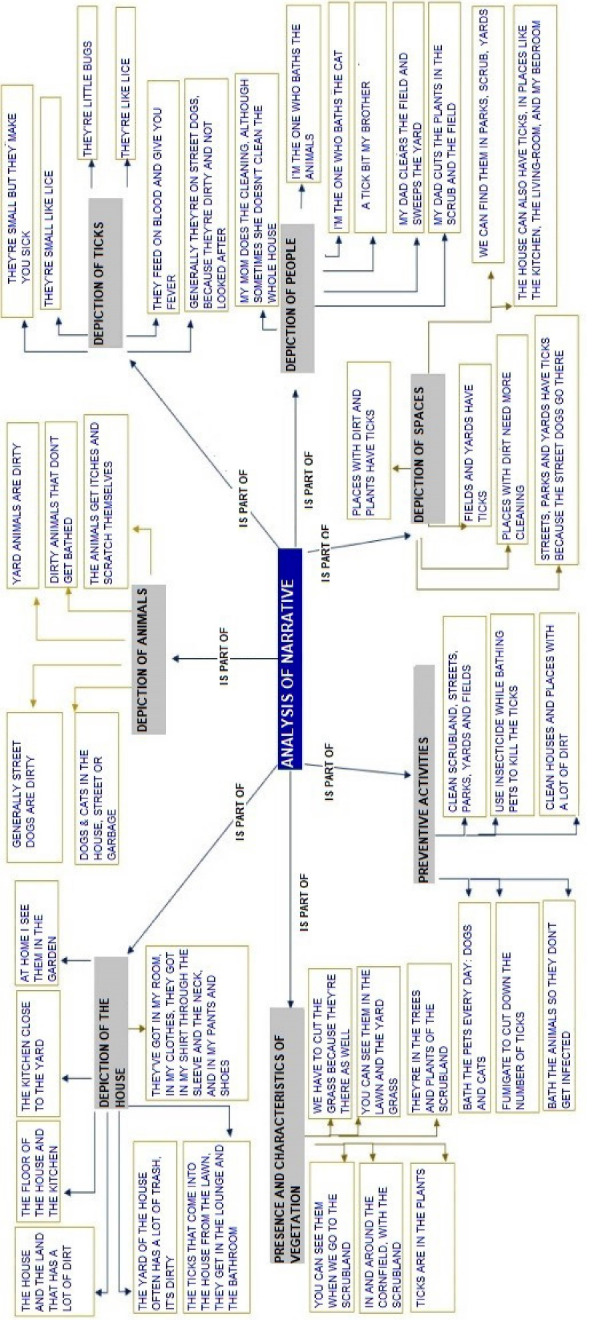
Qualitative categories from an analysis of the participating children’s narratives

 A total of 224 drawings were collected, in which the children depicted the places where they thought they might find ticks and the actions they would take to prevent them from being there. In this way, the perception of individual risk was evaluated and the context in which the children live could be visualized. 99 of the drawings included texts related to ticks and their environment. The drawings and texts were codified using seven categories that consider environmental elements (representations of space and houses, as well as the presence and characteristics of vegetation), actors (significant people) and cognitive aspects (representations of, and beliefs about animals, particularly ticks, and preventive measures) (Table 1 and Figure 1).

**Table 1:** Categorization of children’s drawings

**Depiction **	** Category**	**Sub-category **		**%**	**N**
Space	House			38	85
	Yard or lot			37.5	80
	House and lot			15.2	34
	Agricultural or farming context			5.3	12
	Others			5.8	13
	**Total**				**224**
House	Walls	Concrete		90.6	163
		Traditional		1.1	2
		Mixed		8.3	15
	Roof	Tiled		51.7	93
		Thatch/Huano		12.2	22
		Concrete		34.3	62
		Mixed		1.7	3
	Windows	Yes		86.7	156
		No		13.3	24
	Doors	Yes		95	171
		No		5	9
	**Total**				**180**
Presence and characteristics of vegetation	Yes (206)	Low		56.8	117
		High		43.2	89
	No (18)				
	**Total**				**206**
Animal	Part of transmission cycle	Pets	Dogs	50	113
			Cats	9.8	22
		Domestic farm animals	Horses	2.2	5
			Cows	10.2	23
			Goats	0.4	1
			Bulls	6.6	15
	Not part of transmission cycle	Domestic farm animals	Poultry	9	2
			Pigs	3.1	11
		Wild	Rabbits	1.3	3
		Others	Giraffes	0.8	2
			Fish	1.3	3
			Butterflies	1.3	3
			Birds	9	20
	**Total**				**224**
Ticks	Inside the house			4.6	7
	Outside the house			56.6	134
	Inside and outside the house			6.6	10
	On animals			17	31
	Various places			13.8	25
	**Total**				**207**
People	Boys			84.5	136
	Girls			4.3	7
	Adults			6.7	11
	Old people			1	2
	**Total**				156
Preventive activities	Yes (111)	Bathing the dog		56	62
		Sweeping the yard		12.6	14
		Sweeping the house		9	10
		Spraying with chemical product		10	11
	**Total**				**97**
	No (112)				112

 What follows is a breakdown of the elements which were identified in each category, integrating the drawings and the narratives, and including some of the most representative works, as well as the voices of children who took part.

###  Environmental elements 

####  Representation of space

**Figure 2:**
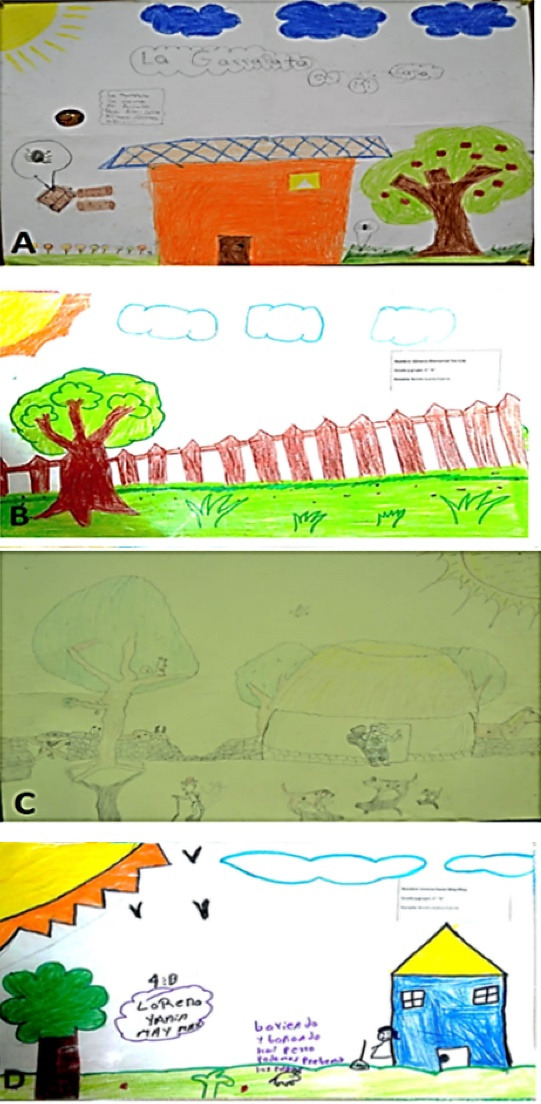
Drawings of external spaces and houses. (A) the house, (B) the lot, (C) Traditional house and (D) Concrete house

 As for the environmental context that allows contact with the phenomenon, the children considered several physical spaces, with 99.1% (222) representing sites outside the dwelling, and only 0.9% (2) drawing the dwelling from the inside. In turn, these external spaces could be categorized into 5 contexts according to the most representative elements: “house”, “yard or plot”, “house and plot”, “agricultural or livestock environment” and “other”. Thirty-eight percent (85) presented the house as the main setting; 35.7% (80) also represented the house but included the yard or lot; 15.2% (34) depicted only the yard or lot; 5.3% (12) drew an agricultural or livestock environment, including cornfields, scrub or a ranch; and finally, 5.8% (13) depicted various scenarios (schools, undeveloped plots, gardens, ruins, hills, parks and forests). Thus, the sites associated with the presence of ticks are primarily within their external environment, in places such as work areas (e.g. the cornfield, the forest) but also close to the housing unit such as in yards and lots, even in recreational or public spaces such as town streets and parks (Figure 2 and Table 1).

*“We can find ticks almost everywhere, like in the scrub” *(Paola, 4^th^ grade).

*“Ticks come from the scrub and sometimes if our puppies go out in the street they can get on them”* (Delta, 4^th^ grade).

####  Description of the home

 Although the presence of ticks is associated with scenarios outside the home itself, 80.4% (180) of the drawings included houses, but shown from the outside, so they were sub-divided into categories depending on the structure and building materials. In these subcategories it was found that 90.6% (163) showed a concrete house; 8.3% (15) mixed houses, that is, they were represented as concrete walls and huano and/or thatched roof; and 1.1% (2) drew a traditional house. As for more specific aspects, the roof was mainly drawn as tiled (51.7%), followed by concrete (34.4%) and, to a lesser extent, huano and/or thatch (12.2% (22). In those drawings that included the interior of the house, the living room or family room, kitchen and bedrooms were identified as vulnerable areas where ticks have been found, mainly in spaces close to the ground (Figure 2, Table 1).

*“Ticks come into the house, in the garden, the kitchen, the bathroom and the living-room” *(Raul, 4^th^ grade).

*“Ticks can be in your yard or in your house or on your dog” *(Rosa, 5^th^ grade).

*“(They are) on your clothes, in your hammock, in the field, in the street, in the store, in the kitchen, on the playing field, on the table, and in your shirt they get into the sleeves or the neck of your shirt, in your pants, in your house and even in your shoes” *(Georgina, 5^th^ grade).

####  Presence and characteristics of vegetation

**Figure 3:**
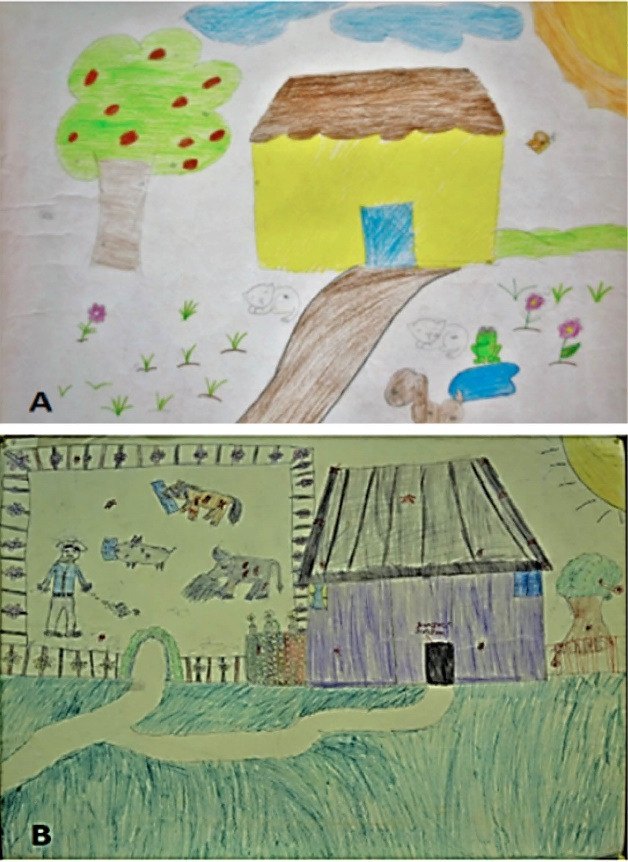
Drawings showing vegetation in the environment. (A) little vegetation, (B) abundant vegetation

 The presence and characteristics of vegetation was another important variable analyzed as part of the environment that exposes one to the problem. Ninety-two percent (206) of the participants included vegetation in their drawings. Of these, 56.8% (117) included it minimally while 43.2% (89) added abundant vegetation to the drawings. Although this dimension provides less data than the others, the information analyzed describes scrubland and cornfields as the prominent forms of vegetation, but the children also identified grass, branches, weeds and lawns, both inside and outside people’s homes (Figure 3 and Table 1).*You can find ticks in plants and in trees.* (Cecilia, 4^th^ grade).

###  Actors

####  Descriptions of people and their activities (preventive, or risky)

 In this section we found that 69.6% (156) of drawings included people. Of these, 84.5% (136) were boys, 4.3% (7) girls, adults 6.7% (11) and the elderly 1% (2) (Table 1).

**Figure 4:**
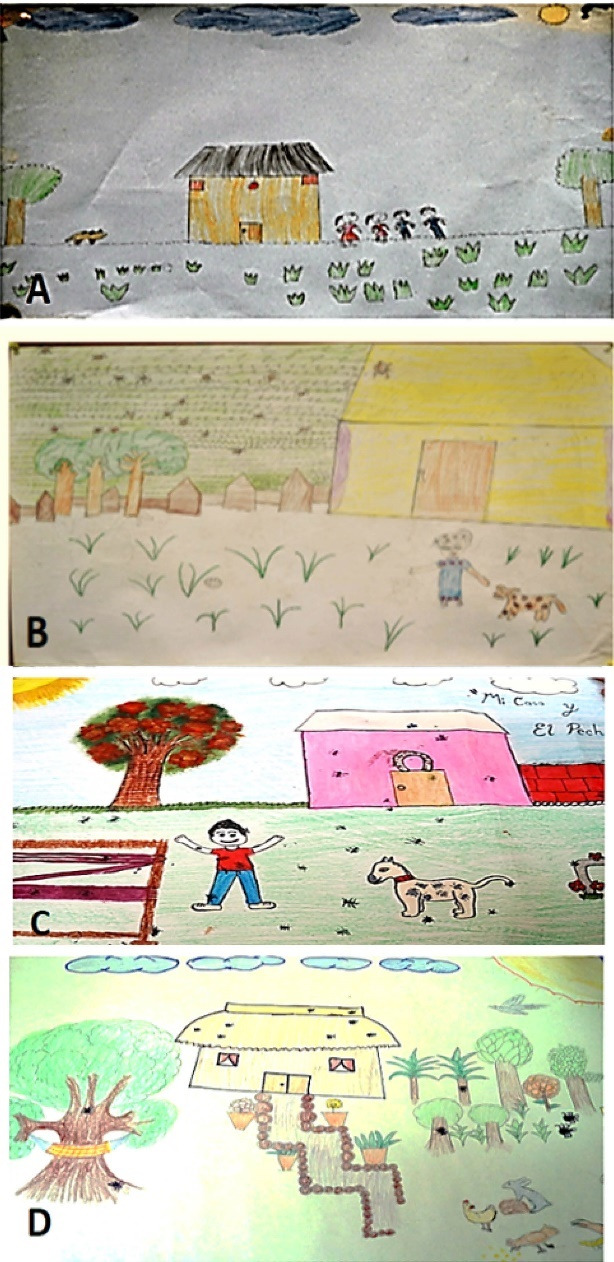
Drawings showing ticks in the environment. (A) Depiction of children, (B) Depiction on older people, (C) Depiction of tick bites on the arms and legs and (D) Depiction of home infestation

 The people are shown doing various activities; most of these are boys and girls (49.5%), moms (37.8%), dads (2.7%) and the elderly (1%) (Figure 4).

 This dimension is particularly important because it underlines two key points. The first one is that the children themselves are directly in contact with ticks in their daily life and they therefore have an active role in the prevention of related diseases. The second is that they identify other people carrying out prevention activities, but also activities that could represent a risk. Thus we have evidence that the participants can learn preventive behaviors indirectly through observation.

*“My Mom is cleaning then house” *(Paola, 4^th^ grade).

*“My Dad is sweeping and I’m bathing my cat because he has ticks” *(Rosa, 5^th^ grade).

*“My Dad is cutting the grass so that the ticks don’t get on my dog; I’m taking fleas off my dog” *(Oswaldo, 4^th^ grade).

###  Cognitive elements

####  Description of animals

**Figure 5:**
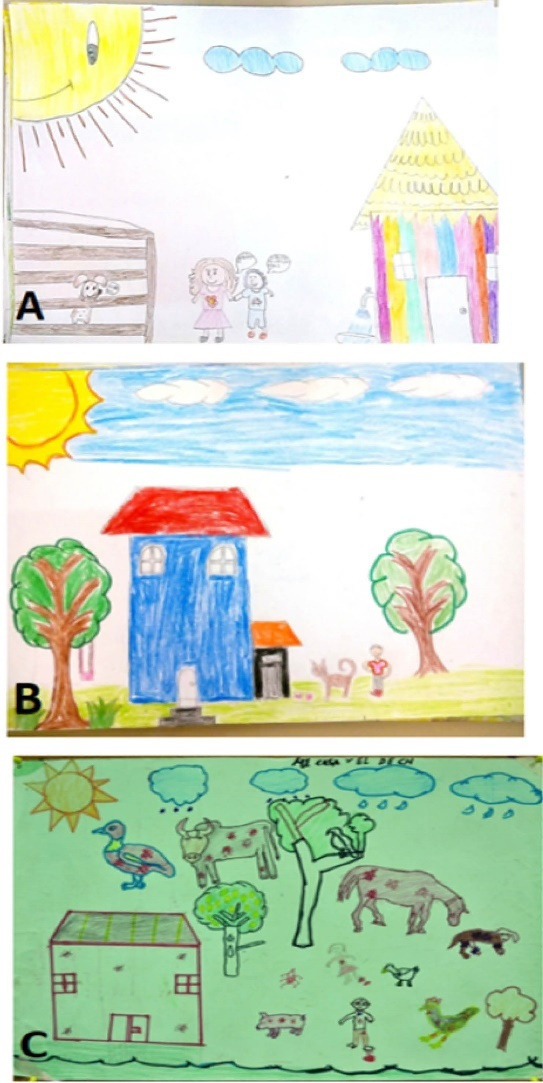
Drawings showing animals in the environment. (A) Depiction of a pet dog, (B) Depiction of a pet cat, (C) Depiction of other animals

 Animals represent a key aspect of the perception of the participating children, since they identify them as the main carriers of ticks, especially “street dogs and cats” stand out but also, to a lesser degree, backyard animals such as pigs. The depiction of animals in the drawings was categorized according to the transmission cycle of tick-associated rickettsial diseases in rural communities; that is, animals that belong to the transmission cycle and animals that do not belong to the transmission cycle. This gave 70% (159) of the drawings that showed animals belonging to the transmission cycle of tick-associated diseases; the principal examples in this category were domestic pets, such as dogs, which appeared in 50% (113) of the total, and cats, which accounted for 9.8% (22). As for domestic farmyard animals with ecological significance for the transmission of diseases, we found that 13.4% (24) of the children had noticed ticks on livestock animals. No wild animals were drawn, although they are part of the transmission cycle (Figure 5 and Table 1).

*“We shouldn’t let cats in the garbage, so that they don’t get ticks” *(Rosalba, 4^th^ grade).

*“The ticks are on the dogs” *(Rosa, 5^th^ grade).

####  Drawings of ticks

**Figure 6:**
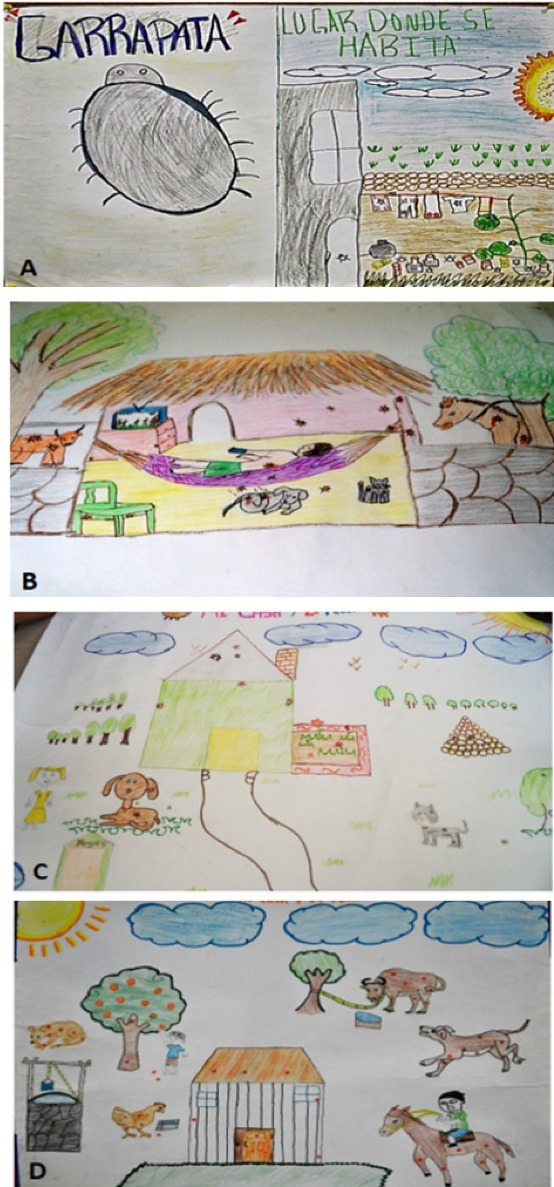
Drawings showing ticks in the environment. (A) Depiction of ticks in the yard and on clothes, (B) Ticks in open spaces, (C) Ticks on dogs and cats and (D) Ticks on animals not linked to the transmission cycle of rickettsiosis.

 Ticks appeared in 92.4% (207) of the drawings. 56.6% (134) were drawn in spaces outside the house, 6.6% (10) both inside and outside, and 4.6% (7) inside the house. The main external places where ticks were depicted were: the ground 17.1% (23), grass or weeds 19.4% (26) and several spaces at the same time 23.8% (32), including trees, walls, etc. As for the drawings that showed ticks inside the house, 2.2% (5) were practically all over the house; while 0.4% (1) had ticks drawn on walls, and 1 on both roof and walls (Figure 6 and Table 1).The perception of these hematophagous ectoparasites is a result of observation, learning and beliefs of the participants, since they see them as small insects that cause particular reactions in both animals and humans, including fever and general discomfort. On the other hand, they also identify them as an insect which can be dangerous for their environment and for people, in that it can bite you, especially on the legs. This was shown in 61.5% (24) of the drawings. Some of the comments mentioning ticks were as follows:

*“I have to take the ticks off when they get on my clothes” *(Lenny, 4^th^ grade).

*“It’s bad if a tick bites you - you can get sick” *(Henry, 4the grade).

*“Ticks suck your blood and give you a temperature” *(Paola, 5^th^ grade).

####  Representation of preventive activities

**Figure 7:**
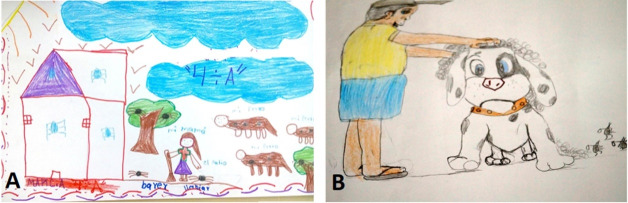
Drawings showing ticks in the environment. (A) Depiction of sweeping the yard, (B) Depiction of a boy bathing his dog

 As far as preventive activities are concerned, 49.8% (111) of the drawings included some (Figure 7 and Table 1). The main activity depicted in the drawings is bathing the dog, which is shown in 56% (62) of the pictures.

*“It’s better to bath them every day, so they feel better, and don’t get disinfectant in their mouths, look after their health and their digestions so they can grow up and have a better life, and keep them in places where there aren’t any ticks so they can sleep better when they get dry” *(Rodolfo, 5^th^ grade).

*“You can get rid of ticks by bathing your dog and the cats, and by weeding and sweeping your house, and taking out the animals from inside your house. Otherwise the ticks will stay” *(Carmen, 4^th^ grade).

 Sweeping the yard or other areas of the house appeared in 12.6% (14) of the drawings, while weeding and clearing fields figured less frequently, at 1% (1) each.

*“2 kinds of things to do to prevent them are cleaning the fields so there aren’t any ticks, and second in animals, bathing the dog every day to get rid of ticks from the dogs and sweeping the yard so that there aren’t any ticks” *(Andrea, 5^th^ grade)

*“You have to check the house, sweep the yard and wash the floor because ticks get in” *(Gema, 5^th^ grade)

*“To avoid ticks we mustn’t throw trash on the floor or bring in dogs, because they can get covered in ticks in the park” *(Oliver, 4^th^ grade).

 One of the activities frequently mentioned in the texts is spraying chemical products, either inside or outside the house, and even on pets.

*“Ticks make you sick, they can kill you and if you spray them with Baygon [insecticide] it kills them and goodbye ticks, they’re dead” *(Gonzalo, 4^th^ grade)

*“Fumigating all the houses and all the yards and the branches and tying up big dogs and small dogs to stop them bringing ticks into the house, and keep the yards clean” *(Reynaldo, 5^th^ grade)

*“What I’d do is buy a detergent that kills them to spray on the dogs and the trees” *(Manuel, 4^th^ grade).

 Finally, some of the texts say that when there are ticks the only option is to kill them.

*“I kill them with my shoe, I kill them with poison, I kill them with my catapult, I kill them with floor cleaner, I kill them with a book, kill them so they don’t bite me and get rid of them so they don’t bite my hand” *(Georgina, 5^th^ grade).

*“To get rid of ticks, sweep them up, throw them away, burn them” *(Daniela, 5^th^ grade).

## Discussion

 The prevention of vector-borne diseases requires that people adopt behaviors in their daily lives that allow them to avoid coming into contact with pathogen-transmitting ectoparasites, or even to eliminate them. This education can take place in various ways - formally or informally, directly or indirectly - but in the vast majority of cases it essentially entails social learning; in other words, learning that occurs through observation of our social environment and interaction with other people.^24^ Therefore, in order to work on prevention of this type of disease, it is important to go back to the socio-environmental and personal factors described by Bandura.^21^

 The health intervention that formed the basis for this work considered these factors when working with children. It was a constant feature of the activities that they should show the participants the importance of “noticing” their own habitat and immediate surroundings, and how they could find ticks in them. This demonstrates a capacity for foresight that allows children to think ahead and rehearse in their minds the consequences of various types of actions. The intervention study also focused on the role that people around children can play in the prevention or non-prevention of TB-rickettsiosis, highlighting the importance of their participation, since this type of problem requires systemic interventions. It was important to show the actions that people close to them are taking to look after them, but also what they are failing to do. Other studies have shown the need for realistic actions that require adult intervention. For example, the use of personal protective measures to prevent tick bites (e.g., repellents); removing clothing worn outside when returning home; showering to increase the likelihood of detecting crawling ticks previously hidden under clothing; checking dogs for the presence of crawling ticks when they enter; and prompt removal of ticks attached to humans (or pets) to reduce the risk of pathogen transmission.^25^

 More important than adult actors, though, is the preponderant role that children give themselves as individuals capable of helping in the prevention of rickettsiosis and other tick-borne diseases. They have learned how to do this, and feel able to contribute to efforts to eradicate the problem. In the case presented here, it can be observed that the participating children have developed the basis for an adequate perception of risk regarding the possibility of contracting TB-rickettsiosis, since they have illustrated the four premises that this type of perception requires.

 New forms of intervention need to be created, with different strategies, that provide follow-up to, and reinforcement of previous behaviors. It is necessary to break paradigms and not to close ourselves within very stereotyped forms of intervention. In this field it is necessary to innovate, both in terms of the objectives to be achieved and the actions to be carried out. As part of this innovation it is important to carry out diverse and new forms of evaluation using virtual reality, augmented reality, videogames and smartphone Apps, which is an aspect that is sometimes neglected.^16,26-28^ Other times, even when an evaluation is proposed, it ends up being rushed or not done at all because the project time is running out. In other aspects, indicators such as the number of participants, the number of activities carried out, or knowledge scores are considered; but rarely are strategies employed to analyze learning in detail, much less to verify its consistent use in daily life. In the end, it is true that the clearest indicator in relation to diseases is a statistical drop in incidence, but reaching that level implies a massive effort that is not always possible. It is in this sense that the evaluation was considered to be as important as the intervention itself, so much care was taken to produce a clear analysis of the achievements won with the intervention. This implied the use of strategies which were both appropriate for what was to be evaluated, and also suitable for the population which we were working with. Thus, two important areas in the development of TB-rickettsial disease prevention are diversifying interventions and making appropriate evaluations of them. In both cases, innovative and relevant strategies are required, even though it is more common to work with the adult population because they are considered to be “rational” people, and because they are responsible for other sectors of the population, such as children or the elderly.^28^

 Methodologies based on the elaboration of drawings, can collect the notions and knowledge of the children, since they feel free to capture their ideas through a playful strategy. Thus, it is important that interventions take the work aimed at children more seriously, with strategies that are appropriate for their age. The more they can understand what is happening, the more likely they are to decide to act and in doing so to bring about beneficial changes.

 Reinforcement is intimately associated with successful behaviors, as well as with revisiting aspects of risk perception that remind people of the reason for the practices they have adopted. Working on follow-up programs for learned behaviors is important, but uncommon, as its importance is underestimated. This happens because work of this kind does not seem to be justified when considering the cost-benefit balance. Financial considerations are important, but it is also needed that everything invested does not end up as a series of efforts with little to show.

 It is not unusual that after an intervention, success is assumed and consequently no further work is done on it. Many behaviors we learn are there to stay, but not all of them. Among the latter are those associated with prevention. If these behaviors are not constantly and regularly reinforced, they are often not sustainable. That is why evaluating what happened after the intervention is important. Studies developed in the daily life to see when and how what is learned in interventions is used is an important kind of studies to be done.

 Due to the nature of the study carried out, the results are not directly generalizable to other populations, but the experience is transferable to populations with similar characteristics. That is, the information can be used to be used or to generate possible interventions bases on the experience carried out.

 It is fundamental that the elaboration of health promotion public policies considers the work with children as important social subjects in the development of healthy habits. For this reason, such policies should not only be psychoeducational, but should include strategies to contribute to supporting the use, in daily life, of what has been learned. And should include strategies considering the age of participants because a lot of psychoeducational programs are adult centric, not only in the interventions but also in the strategies to evaluate what is done.

## Conclusion

 School children have structured and adequate ideas regarding the risk factors that expose them to diseases associated with ticks, as well as their preventive strategies and recognition through symptomatology, which indicates that educational interventions aimed at this population group have a positive impact in the perception and performance that children generate and adopt. It became evident that working with children is fundamental, to the extent that we consider them capable subjects and not child-adults or individuals with little capacity for thought, otherwise we will be losing the opportunity to achieve transformational change. Children tend to be active, committed and supportive, yet we often suppress these characteristics with our actions, instead of taking advantage of them to bring about significant changes in their well-being and that of everyone else.

## Acknowledgements

 To families, municipal authorities, teachers and children from Teabo for participating in the study.

## Competing Interests

 The authors declare no competing interests.

## Ethical Approval

 The Research Ethics Committee of the Regional Research Center of the Autonomous University of Yucatán (Merida, Yucatan, Mexico) approved the ethical statements of this work, as a goal of project CIE-010-1-14.

## Funding

 This research study was funded by W.K. Kellogg Foundation with Grant Number 2330- P6006463.
